# To what extent do general practitioners involve patients in decision-making? A systematic review of studies using the OPTION-instrument

**DOI:** 10.1017/S1463423625100303

**Published:** 2025-07-31

**Authors:** Dirk T. Ubbink, Fadi Shamoun, Steyn Heuvelsland, Faridi S. van Etten-Jamaludin, Eva E. Bolt

**Affiliations:** 1 Department of Surgery, Amsterdam University Medical Center at the University of Amsterdam, Location AMC, Amsterdam, The Netherlands; 2 Faculty of Medicine, University of Amsterdam, Amsterdam, The Netherlands; 3 Research Support Medical Library, Amsterdam University Medical Center at the University of Amsterdam, Location AMC, Amsterdam, The Netherlands; 4 Department of General Practice, Amsterdam University Medical Center at the University of Amsterdam, Location AMC, Amsterdam, The Netherlands

**Keywords:** General practitioners, OPTION-instrument, shared decision-making, systematic review

## Abstract

**Aim:**

This systematic review aimed to analyze studies assessing the extent to which General Practitioners (GPs) engage patients in the decision-making process during consultations.

**Background:**

Shared Decision Making (SDM) stands at the core of patient-centred care, particularly in primary healthcare, where a diverse array of medical decisions transpires. In a 2015 systematic review summarizing studies on the Observing Patient Involvement in Decision Making (OPTION) instrument to assess SDM objectively across healthcare settings, a notable dearth of patient involvement was observed.

**Methods:**

A comprehensive literature search encompassing three digital databases was conducted up to November 2023. Inclusion criteria focused on studies employing a comparative study design, centric to primary healthcare, and utilizing the OPTION-5 or -12 instrument to gauge SDM levels. Two investigators independently performed study selection, risk of bias assessment, and data extraction using a list of predefined variables, with discrepancies resolved by a third reviewer. PROSPERO registration-ID: CRD42023475419.

**Findings:**

Initially, harvesting 447 articles, our review retained 29 studies published between 2003 and 2022. Mean age of GPs was 45.5 (range 33–53) years. Reported baseline OPTION scores varied between 1.5 and 57.2 on a 0–100-point scale, with a median score of 16. Following SDM interventions, OPTION-scores increased significantly to a median of 28.5, range 16–83.

**Conclusion:**

The overall level of SDM among GPs remains relatively low and has shown minimal improvement over the past decade. However, interventions promoting SDM appear to enhance patient involvement levels. This underscores the necessity for increased education and tools, directed at GPs and patients, to foster and elevate the practice of SDM.

## Introduction

Several decades ago, shared decision-making (SDM) emerged as a pivotal principle to enhance patient participation in medical decision-making (Charles *et al.*, 1997; Brody, 1980). In contemporary healthcare, this method of care stands as the cornerstone of patient-centred care (Stiggelbout *et al.*, 2015; Menear *et al.*, 2018) where patients and healthcare providers collaboratively weigh the pros and cons of treatment options, leveraging the best available evidence to reach decisions aligning with the patient’s preferences and circumstances (Elwyn *et al.* 2012; Chambers, 2023). SDM, particularly when complemented by decision aids, yields numerous patient benefits, including heightened satisfaction with the decision-making process, improved knowledge about disease and treatment options, more accurate risk perception, and more fitting treatment choices, all without adverse impacts on health outcomes (Stacey *et al.*, 2024, Bruch *et al.*, 2024).

Despite the supportive evidence for SDM, a 2015 review showed that the level of actually observed patient involvement remains seemingly low (Couët *et al.*, 2015), as indicated by the Observing Patient Involvement in Decision Making (OPTION) instrument. This tool, developed in 2001, exists in 12-item and revised 5-item versions. It is widely utilized as one of the best means to assess patient involvement objectively. Independent observers employ this tool, analyzing audio or video recordings or transcripts of consultations (Barr *et al.*, 2015; Elwyn *et al.*, 2005).

Primary care emerges as an apparent domain for SDM implementation (Elwyn *et al.*, 1999), given the extensive service utilization, diverse health concerns, multitude of daily medical decisions encountered, and the frequent availability of more than one treatment options in this setting (Van der Horst *et al.*, 2023). In the Netherlands, for example, the Federacy of Patient Organisations (PFN) and the Dutch Society of General Physicians (NHG) have been promoting SDM among GPs through a national campaign and e-learnings. However, patient engagement in decision-making within general practitioners’ (GPs) offices appears to be no better than when observed in outpatient clinical settings (Couët *et al.*, 2015).

With the escalating prominence of SDM in recent years (Van der Weijden *et al.*, 2022; Agoritsas *et al.*, 2015), it is plausible that additional evidence concerning SDM application, particularly among GPs, has surfaced since the previous 2015 review. These studies potentially demonstrate increased levels of SDM.

Consequently, we systematically reviewed current literature assessing the extent to which GPs engage patients in the decision-making process during GP encounters, employing the OPTION instrument to measure this involvement. Moreover, we studied factors potentially influencing the level of SDM. As SDM is currently acknowledged as an essential principle in modern, high-quality medicine, the results of this review may help GPs to better involve their patients in the decision-making process and stimulate the implementation of SDM in primary healthcare.

## Methods

This systematic review adhered to the PRISMA guidelines (Page *et al.*, 2021), and was registered in the PROSPERO database under the identification CRD42023475419.

### Search strategy and study selection

A clinical librarian (FvE) helped with conducting the literature search, targeting MEDLINE, Embase, and the Cochrane Library databases from 2014 to September 2023. Studies predating 2014 and meeting our inclusion and exclusion criteria were sourced from a previous systematic review (Couët *et al.*, 2015). Key search terms employed included ‘Shared decision-making’, ‘OPTION-5’, ‘OPTION-12’, and ‘Patient involvement’. Table S1 shows the comprehensive outline of the search strategy. Additionally, the reference lists of pertinent studies underwent scrutiny for relevance. To make sure no relevant studies were overlooked, we also exchanged the harvest of our study search with the colleagues from Spain and the Netherlands who were conducting a general update (PROSPERO CRD42022332231) of the Couët review (Couët *et al.*, 2015).

Two reviewers (SH, FS) independently evaluated study eligibility. Any disparities in assessment were resolved through discussion or, when necessary, consultation with a third reviewer (DU).

### Inclusion and exclusion criteria

Studies were screened for eligibility and included if they utilized observational or experimental study designs employing the OPTION-5 or OPTION-12 instruments to gauge patient involvement objectively in the decision-making process. The study population was restricted to patients receiving care from general or primary care practitioners. Studies utilizing simulated patients or consultations were excluded from this review, as well as those including different healthcare providers and studies in which the data of general practitioners were not reported separately. Additionally, grey literature, abstracts, study protocols, and articles lacking original data were excluded from consideration.

#### Quality assessment

The methodological quality (risk of bias) within the included studies was evaluated based on checklists appropriate for the study designs as reported by the authors. These checklists were obtained from the Cochrane collaboration website (Cochrane, 2024). One reviewer completed these checklists, and a second reviewer independently verified the data entry.

### Data extraction

Study data extraction involved a predefined list of variables, encompassing a) Study characteristics (author, publication year, study design, number of patients included); b) Consultation duration; c) Physician attributes (age, experience duration, prior SDM-education); d) Patient demographics (age, gender, diagnosed disorder); and e) OPTION-score outcomes, both at baseline and following any interventions. One reviewer performed the data extraction, crosschecked by another reviewer. Again, any discrepancies were resolved through discussion involving a third reviewer.

### Data analysis

Analysis of the study characteristics and outcomes was presented as means with standard deviations (SD), medians with interquartile ranges when suitable, or ranges. The OPTION-scores, encompassing both the 12- and 5-item versions, were represented as percentages of the maximum achievable score. Differences between pre- and post-measurements were conveyed as means or medians with ranges or 95% confidence intervals (CI).

Multivariable linear regression analysis using the backward elimination method was employed to examine the impact of year of publication, type of OPTION-instrument used, consultation duration, and patients’ or physicians’ age on the observed OPTION-scores.

Meta-analysis was planned for the primary outcome (OPTION-score) provided acceptable study and statistical heterogeneity. A random effects model to compensate for inter-study variation was used if I^2^ was >50%.

## Results

### Identified studies

**Figure 1.**
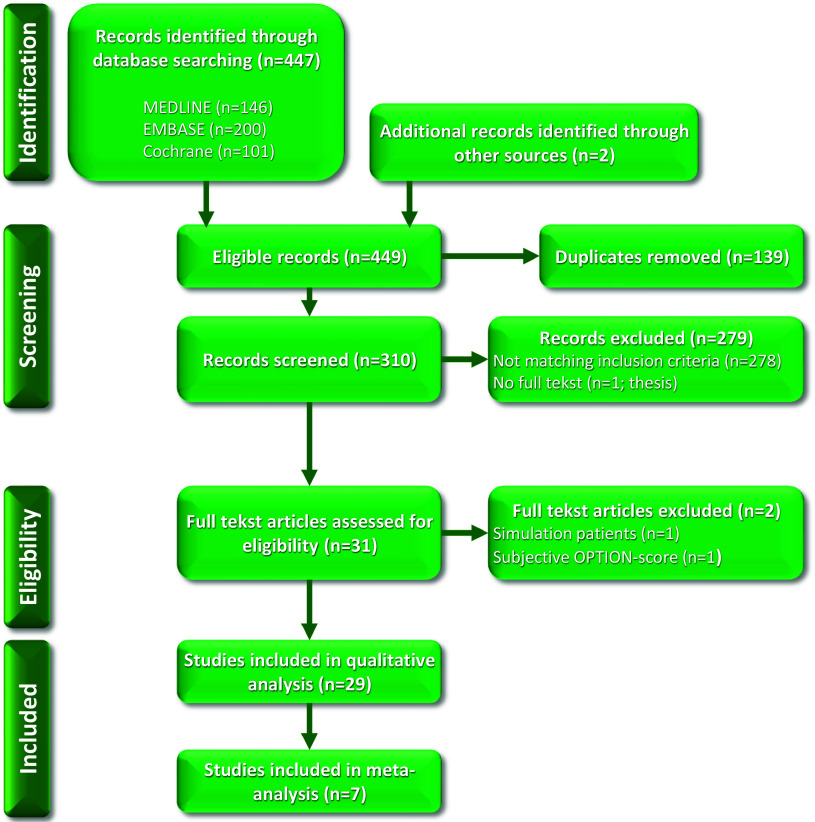
Flow diagram of the selection process.

The search strategy yielded 447 articles. After exclusion of duplicates, 310 publications remained. By reviewing the titles, abstracts, and full texts, 29 papers were included for analysis (Figure 1).

### Study characteristics

Characteristics of the 29 included studies are shown in Table 1. Studies were published between 2003 and 2022. Seventeen were conducted in Europe, six in the USA, two in Australia, two in Canada, and two in Asia. In these 29 studies, GPs mostly discussed treatment options (medication, lifestyle, coaching, psychotherapy) for various conditions, such as diabetes, hypertension, respiratory infections, osteoporosis, obesity, depression, cardiovascular or oncological disorders, as well as screening for lung cancer or Down syndrome. Some studies focused on a single diagnosis, while others included all patients visiting a GP. Consultations were first or routine check-ups at their office, home visits, or specifically to discuss treatment options.

**Table 1.** Study characteristics

Author	Design	Country	OPTION- instrument	N patients / consultations	Patient age (mean, SD or range)	N physicians
Kunneman *et al.*, 2022	Comp	USA	12	350	60 (SD 12)	99
Den Ouden *et al.*, 2022	Obs	Netherlands	5	27	71 (SD 5.6)	9
Chen *et al.*, 2020	Obs	China	5	209	64.4 (SD 8.6)	10
Jackson *et al.*, 2020	Obs	USA	5	105	66.1 (range 46–81)	11
Le Roux *et al.*, 2020	Obs	UK	5	45	47.8	?
Lee *et al.*, 2020	Obs	Malaysia	12	199	57.5 (range 18–87)	31
Misra *et al.*, 2019	Obs	USA	12	24	50.5 (range 32–77)	8
Meijers *et al.*, 2019	Obs	Netherlands	12	100	49 (SD 16)	29
Muscat *et al.*, 2019	Obs	Australia	5	20	75.3	8
Bakhit *et al.*, 2018	Obs	Australia	12	36	36 (range 18–77)	19
Brenner *et al.*, 2018	Obs	USA	12	14	63.9 (SD 5.1)	10
Kölker *et al.*, 2018	Obs	Germany	5	79	54.7 (SD 14.8, range 23–93)	24
Menear *et al.*, 2018	Obs	Canada	12	114	53.5 (SD 17.3)	114
Dillon *et al.*, 2017	Comp	USA	5	40	55.7 (SD 14.7)	26
Sanders *et al.*, 2017	Comp	Netherlands	12	175	45 (SD 14)	42
Dicé *et al.*, 2016	Obs	Italia	12	168	7.0 (SD 3.1)	15
Hirsch *et al.*, 2012	Obs	Germany	12	40	n.r.	15
Sonntag *et al.*, 2012	Obs	Germany	12	58	57	10
Montori *et al.*, 2011	Comp	USA	12	100	67 (range 50–84)	72
Gagnon *et al.*, 2010	Obs	Canada	12	128	29 (SD 3)	41
McKinstry *et al.*, 2010	Comp	UK	12	106	n.r.	19
Weiss and Peters, 2008	Obs	UK	12	117	n.r.	12
Goss *et al.*, 2007	Obs	Italy	12	235	45 (SD 14, range 18–70)	6
Edwards and Elwyn, 2006	Obs	UK	12	68	n.r.	8
Loh *et al.*, 2006	Obs	Germany	12	20	n.r.	9
Siriwardena *et al.*, 2006	Obs	UK	12	63	n.r.	36
Elwyn *et al.*, 2005	Obs	UK	12	186	43	21
Elwyn *et al.*, 2004	Comp	UK	12	352	Range 45–65	20
Elwyn *et al.*, 2003	Obs	UK	12	186	43.3 (20.6, range 0.33–83)	21

Comp = comparing OPTION-scores between different groups.

Obs = one-time assessment of OPTION-score.

n.r. = not reported.

Twenty-three studies were categorized as having an *observational* design, as these scored the observed level of patient involvement only once in a single patient group. Six studies were deemed *comparative*, either before and after the introduction of an SDM-training or communication aid for physicians, or a decision aid for patients. In one study, consultations via telephone were compared with face-to-face consultations (McKinstry *et al.*, 2010). In three studies, the OPTION-12 or the OPTION-5 instrument was tested after translation into the native language (Goss *et al.*, 2007; Hirsch *et al.*, 2012; Kölker *et al.*, 2018). In these studies, as well as the in study by Edwards et al. (Edwards and Elwyn, 2006), the GPs had received some SDM-training before.

It should be noted that in some cases our classification of the study design differed from the design as reported by the study authors; i.e. the study by Den Ouden et al. (Den Ouden *et al.*, 2022) (cluster-RCT testing a decision aid for patients with type-2 diabetes but scoring OPTION-5 only once), Bakhit et al. (Bakhit *et al.*, 2018) (observational study whether SDM occurs in consultations for acute respiratory infections, nested within a cluster-RCT of decision aids), and Meijers et al. (Meijers *et al.*, 2019) (‘observational’ study but comparing OPTION-scores between 2007 and 2015). In the study by Siriwardena et al. (Siriwardena *et al.*, 2006) the OPTION-scores were rated during a consulting skills examination and compared between those who failed or passed the exam, but no SDM-intervention was done. Hence, it was categorized as an observational study.

Audio-recordings with or without transcriptions were used in 20 studies, one study used notes of observations, another observed the live consultations, and the remaining seven studies used video-recordings to rate the level of SDM in the consultations. Out of these 20, 7 used the OPTION-5 instrument to rate the level of SDM.

### Participant characteristics

The number of patients (i.e., consultations) recruited in each study ranged from 14 to 352, with a mean age varying between 29 and 71 years. Overall, slightly more (60.3%) females were involved. One study focused on children, with a mean age of 7.0 years (Dicé *et al.*, 2016).

The number of general physicians who were rated in the studies ranged from 8 to 114, with a mean age varying from 32.9 to 52.7 years, while their mean years of experience ranged from 3.0 to 19.1 years.

### Risk of bias assessment

Table 2 shows the risk of bias assessment of the included comparative studies. Overall, study quality was moderate to good. Obviously, blinding of patients and physicians was hardly possible due to the type of intervention, as physicians knew whether they had utilized an SDM-training or a communication aid, and patients were aware of having used a decision aid.

**Table 2.** Risk of bias in the included comparative studies

Author	1	2	3	4	5	6	7	8	9	10
Kunneman *et al.*, 2022	**+**	**+**	–	**+**	**+**	**+**	**+**	**+**	**+**	**+**
Den Ouden *et al.*, 2022	**+**	**+**	–	**+**	**+**	–	**+**	**+**	**+**	**+**
Dillon *et al.*, 2017	**+**	**+**	–	**+**	–	**+**	**+**	**+**	**+**	**+**
Sanders *et al.*, 2017	**+**	**+**	–	**+**	**+**	**+**	**+**	**+**	**+**	**+**
Montori *et al.*, 2011	**+**	**+**	–	**+**	**+**	**+**	**+**	**+**	**+**	**+**
McKinstry *et al.*, 2010	–	–	–	**?**	**+**	**+**	**+**	**+**	**+**	**+**
Elwyn *et al.*, 2004	**+**	**+**	–	**+**	**?**	**+**	**+**	**+**	**+**	**+**
TOTAL	**87.5%**	**87.5%**	**0%**	**87.5%**	**75%**	**87.5%**	**100%**	**100%**	**87.5%**	**100%**

1: Randomization.

2: Allocation concealment.

3: Blinding of patients and physicians.

4: Binding of observers.

5: Baseline comparability.

6: Complete follow-up.

7: Intention to teat analysis.

8: Similar treatments apart from intervention.

9: Reporting bias ruled out.

10: Academic bias ruled out.

For the single-measurement observational studies, risk of bias is summarized in Table 3. In general, the observational studies carried a low risk of bias.

**Table 3.** Risk of bias in the included observational studies

Author	1	2	3	4	5	6
Chen *et al.*, 2020	–	**+**	**+**	**+**	–	**?**
Jackson *et al.*, 2020	**+**	**?**	**+**	**+**	**+**	**+**
Le Roux *et al.*, 2020	**+**	**?**	**+**	**+**	**+**	–
Lee *et al.*, 2020	**+**	**+**	**+**	**+**	**+**	**?**
Meijers *et al.*, 2019	**+**	**?**	**+**	**+**	**+**	**+**
Misra *et al.*, 2019	**+**	**+**	**?**	**+**	**+**	–
Muscat *et al.*, 2019	**+**	**+**	**?**	**+**	**?**	**?**
Bakhit *et al.*, 2018	**+**	–	–	**+**	**+**	–
Brenner *et al.*, 2018	**+**	**?**	**+**	**+**	**+**	–
Kölker *et al.*, 2018	**+**	**+**	**+**	**+**	**+**	**?**
Menear *et al.*, 2018	**+**	**+**	**+**	**+**	**+**	–
Dicé *et al.*, 2016	**+**	**?**	**+**	**+**	**+**	**+**
Sonntag *et al.*, 2012	**+**	**+**	**+**	**+**	**+**	**+**
Hirsch *et al.*, 2012	**+**	**+**	**+**	**+**	**+**	**+**
Gagnon *et al.*, 2010	**+**	**+**	**+**	**+**	**+**	**+**
Weiss and Peters, 2008	**+**	**?**	**+**	**+**	**+**	–
Goss *et al.*, 2007	**+**	**?**	**+**	**+**	**+**	**?**
Edwards and Elwyn, 2006	**+**	**+**	**+**	**+**	**+**	–
Loh *et al.*, 2006	**+**	**+**	**+**	**+**	**+**	–
Siriwardena *et al.*, 2006	**+**	**+**	**+**	**+**	**+**	**+**
Elwyn *et al.*, 2005	**+**	**+**	**+**	**+**	**+**	**+**
Elwyn *et al.*, 2003	**+**	**?**	**+**	**+**	**+**	**?**
TOTAL	**95%**	**59%**	**86%**	**100%**	**91%**	**36%**

1: Adequate definition of study group.

2: Valid patient selection.

3: Blinded scoring of outcomes.

4: Follow-up duration sufficient.

5: Misclassification ruled out.

6: Corrected for confounding factors.

### Study interventions

SDM interventions consisted of introducing patient decision aids for type 2 diabetes (Kunneman *et al.*, 2022), acute respiratory infections (Bakhit *et al.*, 2018), and for postmenopausal women at risk for osteoporosis (Montori *et al.*, 2011), communication tools to activate and coach patients (Dillon *et al.*, 2017), SDM training for GPs (Sanders *et al.*, 2017; Elwyn *et al.*, 2004). Also, OPTION-scores were compared between video and face-to-face consultations (McKinstry *et al.*, 2010) and between 2007 and 2015 performances (Meijers *et al.*, 2019).

### OPTION-scores

OPTION-scores were usually assessed by two raters independently, but inter-rater agreement by calculating a kappa-value was infrequently reported. Final scores from the two raters were determined by consensus or averaged. The scores and differences between groups were reported differently; either by means per group with or without standard deviations, or by mean differences with a 95%CI or a p-value.

Reported OPTION-scores, on a 0-100 scale, are shown in Table 4. In the 21 studies with untrained participants, reported baseline OPTION-scores ranged from 1.5 to 57.2 on a 0– 100-point scale, with a median of 16.0% (mean 17.6%). In all but one of these studies.

**Table 4.** OPTION-scores in observational and comparative studies

Author	OPTION (control) (%; SD or 95%CI)	OPTION (Intervention) (%; SD or 95%CI)	Consultation duration (min; SD or 95%CI)
Bakhit *et al.*, 2018	22.7 (11.5)	38.8 (6.5)*	9 (4–31)
Brenner *et al.*, 2018	5 (0–17)		13:07 (3:48–27:09)
Chen *et al.*, 2020		30 (25–40)	5.13 (3.25)
Den Ouden *et al.*, 2022		83	
Dicé *et al.*, 2016	4.8 (5.67)		16:18
Dillon *et al.*, 2017	23.9	2 4.5	
Edwards *et al.*, 2006	62.8 (42–78)		
Elwyn *et al.*, 2003	16.9 (7.68)		8.2 (4.0)
Elwyn *et al.*, 2004		26.9 (20.8–39.2)*	12.5
Elwyn *et al.*, 2005		15. 9	8.2
Gagnon *et al.*, 2010	19.7 (7.5-38)		6.5 (3.3, 0.75–17.5)
Goss *et al.*, 2007	20.61 (6–54)		11
Hirsch *et al.*, 2012	Less expertise: 32.1 (21.2)	More expertise: 49.32 (17.0)*	
Jackson *et al.*, 2020	27.5 (0–70)		c: 29.4 vs i: 29.9
Kölker *et al.*, 2018		11.84 (11.92)	
Kunneman *et al.*, 2022	17 (15–20)	25 (23–27)*	c: 28 (20–37) vs i: 26 (16–5)
Le Roux *et al.*, 2020	10.7 (9.3, 0–35)		10:21 (2:18-14:39)
Lee *et al.*, 2020	7.8 (3.3)		14.3 (5.75, 4–38)
Loh *et al.*, 2006	14.7		16.6
Mckinstry *et al.*, 2010	Video: 16 (10.8)	Face-to-face: 19 (9.4)	c: 4.6 (5.8) vs i: 9.7 (4.5)
Meijers *et al.*, 2019	2007: 14.1 (6.3)	2015: 22.6 (11.7)*	c: 9.24 (5.0) vs i: 11.28 (4.2)
Menear *et al.*, 2018	12.34 (4.7)		27.6 (12.4)
Misra *et al.*, 2019	57.2 (51.8–62.6)		
Montori *et al.*, 2011	27.3 (30)	49.8 (30)*	c: 9.4 (2.1-58) vs i: 12.4(2.3–27.4)
Muscat *et al.*, 2019	11.3 (0–35)		16 (6–45)
Sanders *et al.*, 2017	23.66 (20.25–27.08)	38.53 (35.31–41.74)*	c: 13.1 (4.5) vs i: 15.8 (6.0)
Siriwardena *et al.*, 2006	Failed: 27.3	Passed: 35.4*	
Sonntag *et al.*, 2012	1.48 (0.17–2.96)		9.17 (1.55–32.54)
Weiss and Peters, 2008	7.9 (6.77–9.14)		median 8.5 (7.3–9.3)

SD: standard deviation; 95%CI: 95% confidence interval.

* significantly higher OPTION-score than in control group.

**Figure 2.**
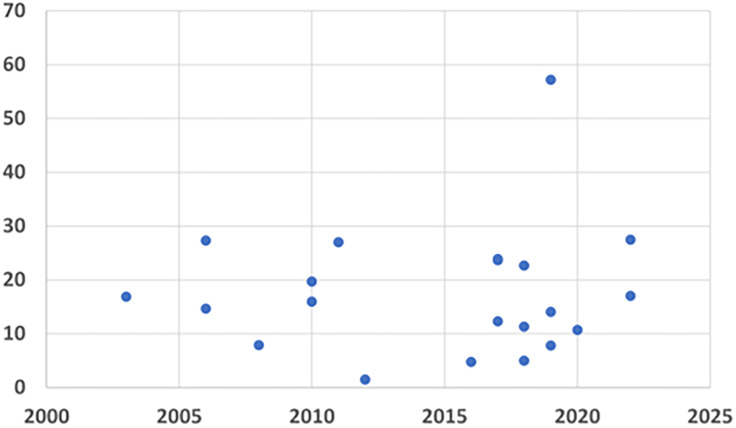
Reported baseline OPTION-scores over time.

OPTION-scores were below 30% (see Figure 2). In the four studies among GPs with some previous SDM-training, (Goss *et al.*, 2007; Hirsch *et al.*, 2012; Kölker *et al.*, 2018; Edwards and Elwyn 2006), baseline OPTION-scores were not significantly higher (median 26.4%, mean 31.8%). However, in the Edwards study, SDM-training for GPs led to a quite high mean OPTION-score of 62.8%, based on 17 purposively selected consultations.

In nine studies with a before-after comparison (Table 1), median OPTION-scores increased from 23.7% (mean 22.6%; range 14.1%–32.1%) to 35.4% (mean 33.5%; range 19.0%–49.3%) after any SDM-intervention. Seven of these studies reported a significant increase in OPTION-scores (Bakhit *et al.*, 2018; Hirsch *et al.*, 2012; Kunneman *et al.*, 2022; Meijers *et al.*, 2019; Montori *et al.*, 2011; Sanders *et al.*, 2017; Siriwardena *et al.*, 2006). Another study, reporting only a 26.9% increase in OPTION-score after SDM-training, also showed a significant improvement (Elwyn *et al.*, 2004).

**Figure 3.**
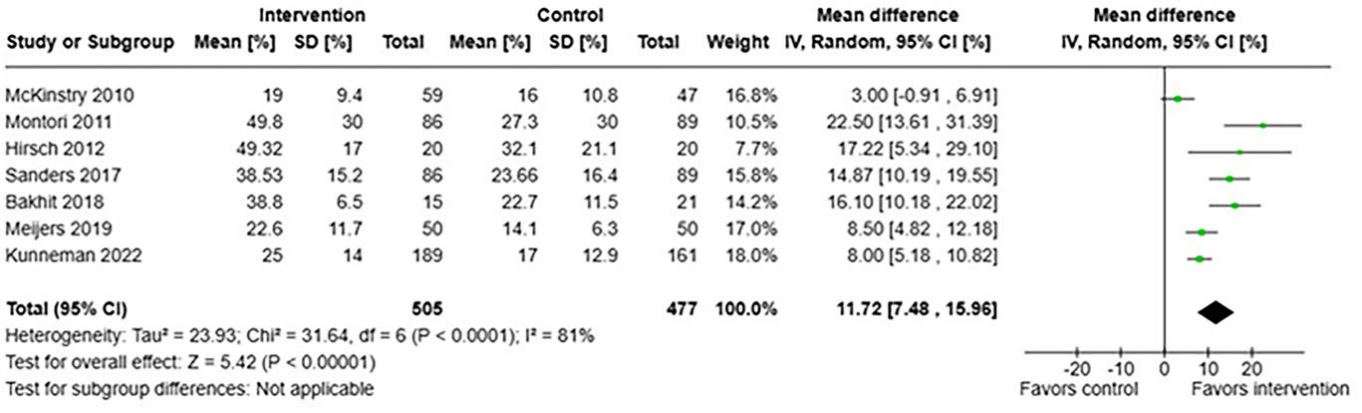
Forest plot of OPTION-scores.

Meta-analysis of the seven studies that reported OPTION-scores before and after an SDM-intervention is shown in Figure 3. When pooled, a significant increase was seen in OPTION-scores after a SDM-intervention: Mean difference was 11.72%, 95%CI 7.48–15.96, albeit with a large heterogeneity. Removing the studies with higher risk of bias (Bakhit *et al.*, 2018; McKinstry *et al.*, 2010) did not change the outcome substantially.

### Consultation duration

Based on the 22 studies reporting on consultation duration, mean duration was 13 mins. (median 10.68, range 4.6–29.4 mins.). Six studies reported consultation durations before and after a SDM-intervention. Before the intervention, mean duration was 15.6 mins. (median 11.25 mins.), which did not differ significantly from the duration after intervention (mean 17.51, median 14.10 mins.)

### Regression analysis

Neither univariable nor multivariable analyses yielded factors significantly influencing the OPTION-scores. In particular, the consultation duration and the year of publication did not influence the OPTION-scores (see Figure 2). Included studies published before 2012 had a mean baseline OPTION-score of 21.7% (SD 15.4) vs. 18.7% (SD 14.4) in those published 2013 or later, i.e. after the previous review by Couët et al. (Couët *et al.*, 2015).

## Discussion

The current evidence from 29 studies included in this systematic review of the literature on the level of SDM in primary care shows that there is still a low level of SDM among general practitioners. This level significantly improved after the introduction of SDM-supporting interventions, such as patient decision aids, question prompts and SDM-education, but still leaves room for improvement. Over time, since the first study published in 2003, the observed level of SDM appears to remain unchanged. The OPTION-instrument is a common and useful way of capturing SDM-behaviour and changes in SDM-skills over time.

In primary care settings, a higher level of patient involvement might be expected. Patients who see their GPs regularly may have developed a higher level of confidence, while GPs may easier invite their patients to share their ideas, concerns and preferences. On the other hand, the presented complaints and illnesses are usually different from those in an outpatient clinical setting. Issues at stake may have a smaller impact on physical health, in contrast with encounters with medical specialists, in which decision-making may be more focused on comparing treatment options.

The stagnating SDM-levels among GPs may be due to still insufficient perceptions of the SDM-model (Torres-Castaño *et al.*, 2024). The SDM-skills did not improve over time, but this may (at least in part) be explained by the fact that the studies were conducted among untrained GPs. However, even studies among GPs with some SDM-expertise (Goss *et al.*, 2007; Hirsch *et al.*, 2012; Kölker *et al.*, 2018; Edwards and Elwyn 2006) did not show significantly higher OPTION-scores. Also, the long-term effects of these SDM-interventions were not investigated and may have been short-lived. Hence, a combination of undergraduate and post-graduate SDM-education and continual SDM-training are likely to yield a more permanent effect (Col *et al.*, 2011; Légaré *et al.*, 2011; Nyamapfene and Merchant, 2023; Elwyn *et al.*, 2003). Simultaneously, patients need to be informed and educated to better participate in this decision-making process (Wagner *et al.*, 2019; Légaré *et al.*, 2012). However, the best way to implement SDM among healthcare professionals in general is still unclear (Légaré *et al.*, 2018). Further research should focus on implementation initiatives and ways to sustain the effect of the interventions.

No effects were seen of the consultation duration on the observed SDM-levels. This agrees with a previous review (Van Veenendaal *et al.*, 2023), which showed that more SDM does not necessarily lead to a longer duration. The patients’ age also did not seem to influence the observed SDM-levels. This is in contrast with current ideas (Schneider *et al.*, 2006). As the studies included in the present review contained mostly middle-aged or elderly participants, our regression analysis may not have been sensitive to a possible association with age.

## Strengths & limitations

This review included and analyzed 29 studies in the primary healthcare setting, which is a substantially higher number than in the previous 2015 review by Couët et al. (Couët *et al.*, 2015), in which 12 out of the included 33 studies addressed primary care. The risk of bias of the included studies was moderate to good.

Limitations of this review include the fact that OPTION-score ratings are operator-dependent, and the interpretation of the items may need calibration for each patient populations. Usually, more than one rater scored the consultations, but only in some studies their inter-rater agreement was assessed. Hence, rating skills and interpretations may have differed across the included studies. Also, two versions of the OPTION-instrument were applied: one version measuring the magnitude (OPTION-5) of the patient involvement by the clinician, and the other the attitude towards patient involvement (OPTION-12). This may have led to diverging outcomes, although the two versions were found to correlate well (Stubenrouch *et al.*, 2016). Despite these possible causes for uncertainty, the impact on our conclusion seems limited as OPTION-scores were generally low across all included studies, irrespective of the type of OPTION-instrument used or patient population studied.

## Conclusion

SDM is considered as an ethical obligation in modern healthcare and seems desirable and feasible in primary healthcare. However, current evidence shows the level of SDM in consultations between patients and their GPs still leaves room for improvement. This improvement is feasible indeed, as SDM-levels were shown to improve significantly with interventions such as decision aids, pre-scripted patient questions, and SDM-trainings. Long-term effects are still unknown and need further research.

This systematic review on the level of SDM in primary care can hopefully contribute to help GPs to better involve their patients in the decision-making process. The evidence from this review can also be seminal for policymakers to stimulate the implementation of SDM in this specific medical realm.

## Supporting information

Ubbink et al. supplementary material Ubbink et al. supplementary material

## Supplementary material

For supplementary material accompanying this paper visit https://doi.org/10.1017/S1463423625100303
