# Supplementary material for: To what extent do general practitioners involve patients in decision-making? A systematic review of studies using the OPTION-instrument
Source: Prim Health Care Res Dev. 2025 Jul 31;26:e67. doi: 10.1017/S1463423625100303 (PMC12455353; doi:10.1017/S1463423625100303)
Supplement: Ubbink et al. supplementary material [file S1463423625100303sup001.docx]

**Supplementary table S1.** Search strategies in MEDLINE, Embase and the Cochrane library

**MEDLINE** (via OVID):

| # | Searches |
| --- | --- |
| 1 | decision making, shared/ |
| 2 | (shared decision* or patient decision* or patient involvement).ti,ab,kf. |
| 3 | 1 or 2 |
| 4 | (option-5 or option-12).ti,ab,kf. |
| 5 | observing patient involvement.ti,ab,kf,mp. |
| 6 | (option* adj3 (instrument* or scale* or score*)).ti,ab,kf. |
| 7 | 4 or 5 or 6 |
| 8 | 3 and 7 |
| 9 | limit 8 to yr="2014 -Current" |

**Embase** (via OVID):

| # | Searches |
| --- | --- |
| 1 | shared decision making/ |
| 2 | (shared decision* or patient decision* or patient involvement).ti,ab,kf. |
| 3 | 1 or 2 |
| 4 | (option-5 or option-12).ti,ab,kf. |
| 5 | observing patient involvement.ti,ab,kf,mp. |
| 6 | (option* adj3 (instrument* or scale* or score*)).ti,ab,kf. |
| 7 | 4 or 5 or 6 |
| 8 | 3 and 7 |
| 9 | limit 8 to yr="2014 -Current" |

**Cochrane Central Register of Controlled Trials:**

| # | Searches |
| --- | --- |
| 1 | (shared decision* or patient decision* or patient involvement):ti,ab,kw |
| 2 | (option-5 or option-12):ti,ab,kw |
| 3 | (observing patient involvement):ti,ab,kw |
| 4 | (option* near/3 (instrument* or scale* or score*)):ti,ab,kw |
| 5 | #2 or #3 or #4 |
| 6 | #1 and #5 |
